# Anti-TNF-alpha agents and endothelial function in rheumatoid arthritis: a systematic review and meta-analysis

**DOI:** 10.1038/s41598-017-05759-2

**Published:** 2017-07-13

**Authors:** Francesco Ursini, Christian Leporini, Fabiola Bene, Salvatore D’Angelo, Daniele Mauro, Emilio Russo, Giovambattista De Sarro, Ignazio Olivieri, Costantino Pitzalis, Myles Lewis, Rosa Daniela Grembiale

**Affiliations:** 1 Department of Health Sciences, University of Catanzaro “Magna Graecia”, Catanzaro, Italy; 2 Rheumatology Department of Lucania, San Carlo Hospital of Potenza and Madonna delle Grazie Hospital of Matera, Potenza, Italy; 3 Centre for Experimental Medicine & Rheumatology, William Harvey Research Institute and Barts and The London School of Medicine and Dentistry, Queen Mary University of London, London, United Kingdom

## Abstract

Rheumatoid arthritis (RA) has been associated with endothelial dysfunction, a pathophysiological feature of atherosclerosis. Our aim was to determine whether TNF-α blockade has a beneficial effect on endothelial function in RA. We performed a systematic review with meta-analysis to evaluate the effect of anti-TNF-α agents on endothelial function in RA patients. MedLine, Cochrane CENTRAL and SCOPUS were searched up to March 2016. Inclusion criteria were: 1) randomised controlled trial (RCT), quasi-RCT, before-after cohort study; 2) including RA patients; 3) treatment with anti-TNF-α medications; 4) evaluating the change from baseline in endothelial function. The search strategy retrieved 180 records, of which 20 studies were included in the systematic review. Pooled analysis using a random-effects model demonstrated a significant improvement in endothelial function following anti-TNF-α treatment (SDM 0.987, 95%CI [0.64–1.33], p < 0.0001). Generalisation of the results of the meta-analysis may be limited due to the presence of heterogeneity (I2 = 82.65%, p < 0.001) and evidence of possible publication bias. Meta-regression showed that endothelial function measurement technique was a significant contributor to heterogeneity. In conclusion, although limited by the methodological quality of the included studies, our meta-analysis suggests that anti-TNF-α treatment may improve endothelial function in RA patients.

## Introduction

Rheumatoid arthritis (RA) is characterised by an excess of cardiovascular diseases (CVD) risk, comparable in magnitude to that conferred by type 2 diabetes mellitus (T2DM)^1^. To explain this phenomenon, a synergy between traditional risk factors and inflammatory disease activity has been proposed^2^. In addition, literature evidence shows that CVD risk factors such as high blood pressure^3^, T2DM^4^, insulin resistance^5, 6^ and dyslipidaemia^7^ are still underdiagnosed and undertreated in RA patients.

Among emerging CVD risk factors, an important role is played by endothelial dysfunction, a pathophysiological condition able to independently predict CVD events in the general population^8^. An impaired endothelial function has been largely demonstrated in RA patients^9^ and could contribute to the progression of atherosclerosis in this population^10^.

The endothelium is the main regulator of vascular homoeostasis^11^. A functional endothelium is essential in maintaining control of arterial tone, coagulation status, and smooth muscle cells proliferation. Conversely, endothelial dysfunction is characterised by an imbalance between vasodilating mediators with anti-mitogenic and anti-thrombogenic activity such as nitric oxide and prostacyclin, and vasoconstricting mediators with prothrombotic, proliferative effects such as endothelin-1^12^. Injury to the vascular endothelium is believed to be a preliminary event in most vascular diseases^13^.

Several techniques have been developed for the invasive and non-invasive assessment of endothelial function in humans. Most of these techniques evaluate endothelial function by quantifying the vascular response to pharmacological or physical stimuli (i.e. acetylcholine, experimental ischaemia). To date, flow-mediated dilatation (FMD), venous occlusion plethysmography (VOP), peripheral arterial tonometry (PAT) and laser-Doppler iontophoresis (LDI) have been largely validated in clinical studies, although each technique has advantages and disadvantages^14^.

Tumor necrosis factor-alpha (TNF-α) is a pleiotropic pro-inflammatory cytokine with a recognised pivotal role in RA pathogenesis. Additionally, pre-clinical and clinical evidence support the role of TNF-α in atherosclerosis. Higher circulating levels of TNF-α are present in CVD patients and TNF-α itself is able to directly impair endothelial function reducing nitric oxide synthase expression and triggering NF-κB activation and reactive oxygen species accumulation in endothelial cells^15, 16^.

Anti-TNF-α therapy, now a cornerstone of RA treatment together with other biologic agents, has been demonstrated to improve cardiovascular outcomes and to reduce several cardiovascular risk factors^17–21^. Some evidence point to a beneficial effect of anti-TNF-α agents on vascular wall physiology raising the possibility that TNF-α blockade may improve endothelial function in RA patients with consequently reduced progression of subclinical atherosclerosis and improvement of arterial stiffness^22^.

However, clinical studies conducted to investigate the effect of anti-TNF-α therapy on endothelial function in these patients have shown inconsistent results generating controversy on this subject. Therefore, the aim of this study was to investigate the medium- to long-term effect of anti-TNF-α biologics on endothelial function in RA patients by a systematic review and meta-analysis of available studies.

## Materials and Methods

### Search strategy

A systematic review of the literature was performed in order to identify the available data on medium- to long-term effect of anti-TNF-α biologic agents on endothelial function in RA patients. For manuscript preparation, we followed the MOOSE (Meta-analysis Of Observational Studies in Epidemiology)^23^ guidelines for reporting systematic reviews and meta-analyses. MedLine (*via* PubMed), Cochrane Central Register of Controlled Trials (CENTRAL) and SCOPUS databases were searched up to March 2016. The main search in MedLine and Cochrane CENTRAL was conducted using the string (“flow mediated dila*” OR “FMD” OR “forearm blood flow” OR “FBF” OR “endothelial dysfunction” OR “endothelial function”) AND (“rheumatoid arthritis”) AND (“infliximab” OR “adalimumab” OR “etanercept” OR “certolizumab” OR “golimumab” OR “anti TNF”). The main search in SCOPUS was conducted using the string TITLE-ABS-KEY ((“flow mediated dila*“ OR “FMD” OR “forearm blood flow” OR “FBF” OR “endothelial dysfunction” OR “endothelial function”) AND (“rheumatoid arthritis”) AND (“infliximab” OR “adalimumab” OR “etanercept” OR “certolizumab” OR “golimumab” OR “anti TNF”)).

Additionally, the keywords “flow mediated dilatation”, “FMD”, “forearm blood flow”, “FBF”, “endothelial dysfunction”, “endothelial function”, “rheumatoid arthritis”, “infliximab”, “adalimumab”, “etanercept”, “certolizumab”, “golimumab” and “anti TNF” were used in different combinations in order to improve the sensitivity of the search strategy. Bibliography of relevant articles was hand-searched for identification of other potentially relevant studies. The search was designed and performed by one author (DM).

### Inclusion criteria and study selection

To be included in the final analysis, studies had to meet the following inclusion criteria:

1. **Study design:** randomised controlled trial (RCT), *quasi*-RCT (trials in which allocation to treatment was made by alternation, use of alternate medical records, date of birth or other expected methods), prospective (before-after) cohort study;
2. **Population:** studies including RA patients;
3. **Intervention:** treatment with anti-TNF-α medications including infliximab (IFX), adalimumab (ADA), etanercept (ETN), certolizumab pegol (CZP), golimumab (GOL) with a follow-up duration ≥2 weeks;
4. **Outcome:** change from baseline in endothelial function assessed by FMD, VOP, PAT, LDI. A short description of these techniques is available in Supplementary Table S1.

Limits: only full-text version published in English language were considered.

**Figure 1:**
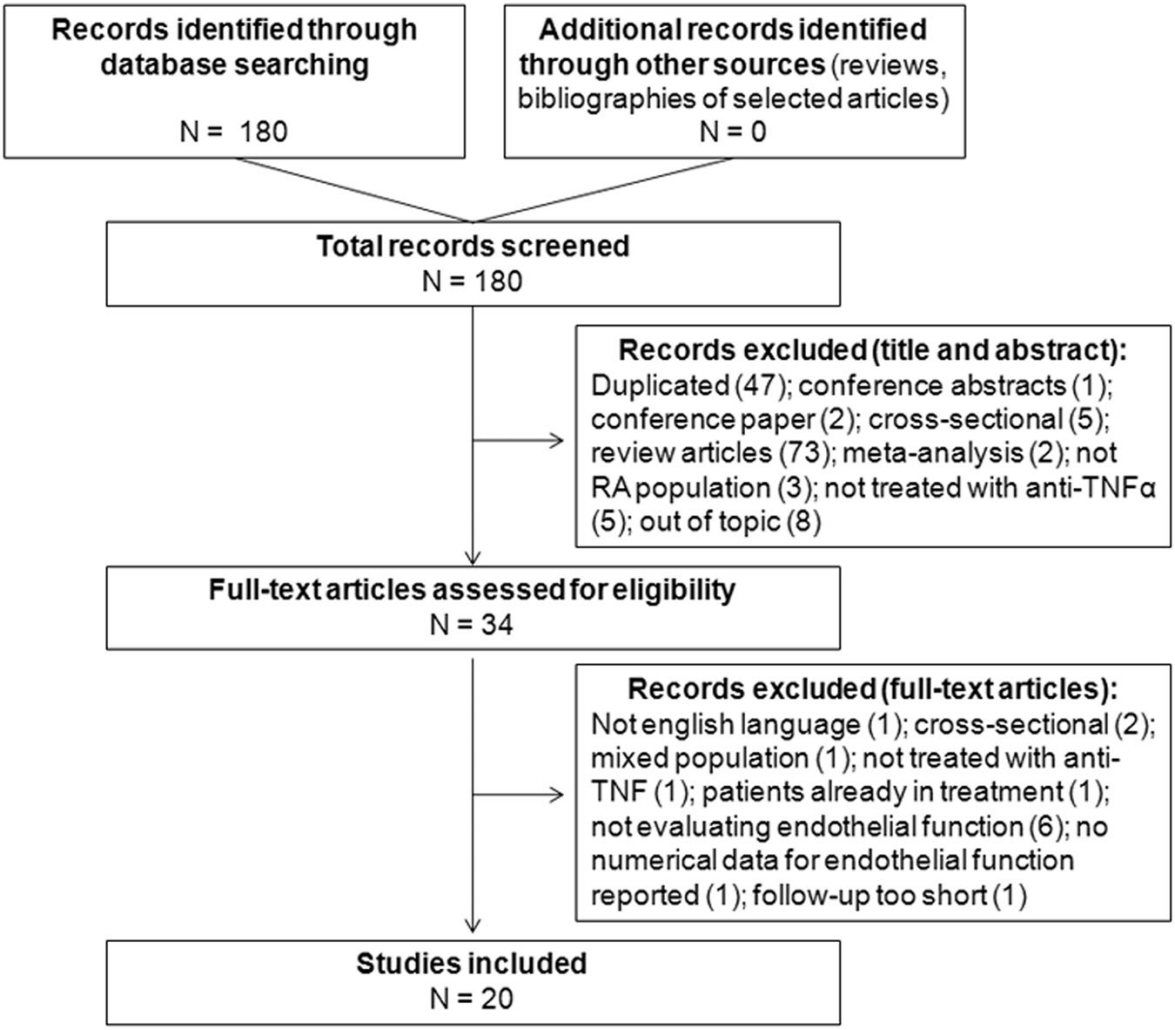
Study selection flow-chart. The process of search and selection of studies is disclosed. Causes of exclusion at each step are described and the number of studies excluded is reported in brackets.

Two reviewers (FU and CL) independently screened titles and abstracts of retrieved records for inclusion in the systematic review. After screening phase, the same two reviewers independently evaluated the selected abstracts and the full-text of these studies to determine eligibility according to the inclusion criteria. Disagreements among the reviewers were resolved by discussion with a third senior reviewer (ER) until reaching a final consensus. A detailed flowchart of the study selection process is depicted in Fig. 1.

### Data extraction

Data extraction was performed by two independent reviewers (FU and CL) and independently verified by a senior reviewer (SD). The following data were extracted from selected studies: first author name, study year, study design, specific anti-TNF-α molecule, control drugs (if any), duration of follow-up, measure of endothelial function before and after treatment and corresponding *p*-value. When reported, endothelial-independent responses (i.e. sodium nitroprusside– or glyceryltrinitrate–stimulated) were not considered in the analysis. For studies in which endothelial function was assessed at more than two time-points, only the last value was considered for the analysis. For studies in which assessment of endothelial function was performed before and immediately after the administration of the biologic agent at different time points, only the baseline value and the last pre-treatment values were considered for the analysis.

### Quality and risk of bias assessment

Quality assessment was performed by two independent reviewers (FU and CL) using the Quality Assessment Tool for Before-After (Pre-Post) Studies With No Control Group proposed by the National Heart, Lung, and Blood Institute - US Department of Health & Human Services (https://www.nhlbi.nih.gov/health-pro/guidelines/in-develop/cardiovascular-risk-reduction/tools/before-after). After scoring each item an overall rate (good, fair or poor) was assigned by each reviewer. Disagreements among the reviewers were resolved by discussion with a third senior reviewer (ER) until reaching a final consensus.

### Data analysis

Data from relevant studies were pooled using the random-effects model that accounts for the expected high heterogeneity. The effect size for endothelial function using pre- and post- anti-TNF-α treatment values was expressed as standardised difference in means (SDM) with 95% confidence intervals (CIs). For studies that provided only median and interquartile range (IQR) values, mean and standard deviation (SD) were calculated by applying the formula by Wan *et al*.^24^ that provides a better estimation than that proposed by Hozo *et al*.^25^. Pre/post correlation was incorporated in order to calculate the covariance of the paired difference for each study. Since the pre/post correlations were not reported in any study, we calculated effect sizes by assuming positive correlations of 0.25, 0.50 and 0.75. These approaches yielded essentially identical results; therefore the findings reported in this paper are based on a standardized correlation of 0.50. Heterogeneity was assessed using the Cochrane Q statistic, the τ^2^ statistic, the H^2^ statistic and the I^2^ statistic^26^; I^2^ values of 25%, 50% and 75% were considered to correspond to low, medium and high levels of heterogeneity, respectively. To evaluate the robustness of results and to identify the potential sources of heterogeneity, we performed *one-study-removed* sensitivity analysis, subgroup analysis and random-effects meta-regression analyses. Publication bias was graphically assessed by visual inspection of the funnel plot and confirmed using the Egger’s regression test^27^. The *trim and fill* method proposed by Duval and Tweedie^28^ was used to calculate pooled estimates after correction for potential publication bias. A two-tailed p-value < 0.05 was considered significant. Statistical analyses were performed by using Comprehensive Meta-Analysis software (CMA; Version 2.0, Englewood, USA). Random effects model meta-regression was analysed in R (version 3.3.2) using the meta and metafor packages.

### Data availability

The datasets generated during and/or analysed during the current study are available from the corresponding author on reasonable request.

## Results

### Search results

The search strategy initially retrieved 180 records (Fig. 1). No additional citations were added by personal search. After screening titles and abstracts, a total of 146 studies were excluded because of search overlap, because dealing with the wrong population/intervention, out of topic, conference contributions or review articles.

Amongst the remaining 34 studies selected for full-text examination, only 20 articles were reviewed in detail and included inthe systematic review. Causes of exclusion are detailed Supplementary Table S2.

### Characteristics of the included studies

Of the included studies, 16 were observational studies^29–44^ and only four^45–48^ were quasi-controlled trials. Characteristics of the studies included in pooled analysis are reported in Table 1 and Supplementary Table S3. The overall quality of the studies was low, with 12 studies receiving a rate of *poor* and 8 of *fair* quality as detailed in Supplementary Table S4.

**Table 1:** Main results of the studies included in the final analysis.

Study, year	Design	N° patients	EF-pre	EF-post	*P* value	Quality
**Technique: Flow mediated dilatation (FMD)**						
**Bilsborough**, 2006	Ob	9	6.5 ± 1.4	8.6 ± 1.5	0.009	Poor
**Bosello**, 2008	Ob	10	7.7 ± 2.8	9.1 ± 2.9	—	Poor
**Capria**, 2004	Ob	10	3.8 ± 3.7	13.9 ± 7.3	<0.05	Poor
**Capria**, 2010	Ob	24	4.6 ± 4.1	13.5 ± 6.0	<0.05	Poor
**Foster**, 2010	Ob	24	2.57 ± 3.36	2.45 ± 3.35	0.29	Fair
**Gonzalez-Juanatey**, 2006	Ob	8	5.8 ± 4.1	8.9 ± 5.7	0.01	Fair
**Gonzalez-Juanatey**, 2012	Ob	34	4.5 ± 4.0	7.4 ± 2.8	<0.001	Fair
**Hurlimann**, 2002	Ob	11	3.2 ± 0.4	4.1 ± 0.5	0.018	Fair
**Irace**, 2004	Ob	10	3.7 ± 1.9	4.2 ± 2.4	—	Poor
**Kerekes**, 2011	Ob	8	7.0 ± 5.9	13.2 ± 5.6	<0.05	Poor
**Maki-Petaja**, 2012	Ob	17	3.54 ± 2.34	6.66 ± 3.17	0.003	Poor
**Park**, 2014	Ob	29	3.8 ± 3.3	5.8 ± 3.4	0.017	Poor
**Sandoo (1)**, 2012	Ob	23	9.4 ± 6.8	12.0 ± 8.1	0.21	Fair
**Sandoo (2)**, 2012	Ob	23	9.4 ± 6.9	12.0 ± 8.1	0.21	Fair
**Sidiropoulos**, 2009	QCT	12	7.0 ± 4.3	9.0 ± 4.9	0.30	Poor
**Spinelli**, 2013	Ob	17	8.25 ± 0.09	8.7 ± 0.06	0.49	Fair
**Tikiz**, 2010	QCT	11	5.2 ± 0.8	7.9 ± 1.3	0.04	Poor
**Technique: Laser Doppler Iontophoresis (LDI)**						
**Foster**, 2010	Ob	24	289.7 ± 166.3	267.0 ± 102.4	0.31	Fair
**Sandoo (1)**, 2012	Ob	23	314 ± 248	248 ± 209	0.01	Fair
**Sandoo (2)**, 2012	Ob	23	319 ± 217	348 ± 309	0.02	Fair
**Technique: Peripheral arterial tonometry (PAT)**						
**Hjeltnes**, 2012	QCT	21	1.94	2.08	0.51	Fair
**Hjeltnes**, 2013	QCT	30	1.94	2.06	0.43	Poor
**Technique: Venous occlusion pletismography (VOP)**						
**Komai**, 2007	Ob	15	36.0 ± 2.2	43.7 ± 2.5	<0.05	Poor

Legend: EF, endothelial function; Ob, observational; QCT, quasi-controlled trial.

The total number of anti-TNF-α-treated patients was 346, of which 61 received Infliximab (IFX), 122 Adalimumab (ADA) and 82 Etanercept (ETN). The remaining 81 patients were treated with IFX or ADA or ETN, but no detailed data were available. There were no patients treated with Golimumab (GOL) or Certolizumab pegol (CZP). Mean age of the patients was 51.8 ± 5.0 years (mean ± SD), while mean follow-up duration was 15.3 ± 11.7 weeks (range 4–52 weeks).

### Pooled analysis of the effect of anti-TNF-α on endothelial function

**Figure 2:**
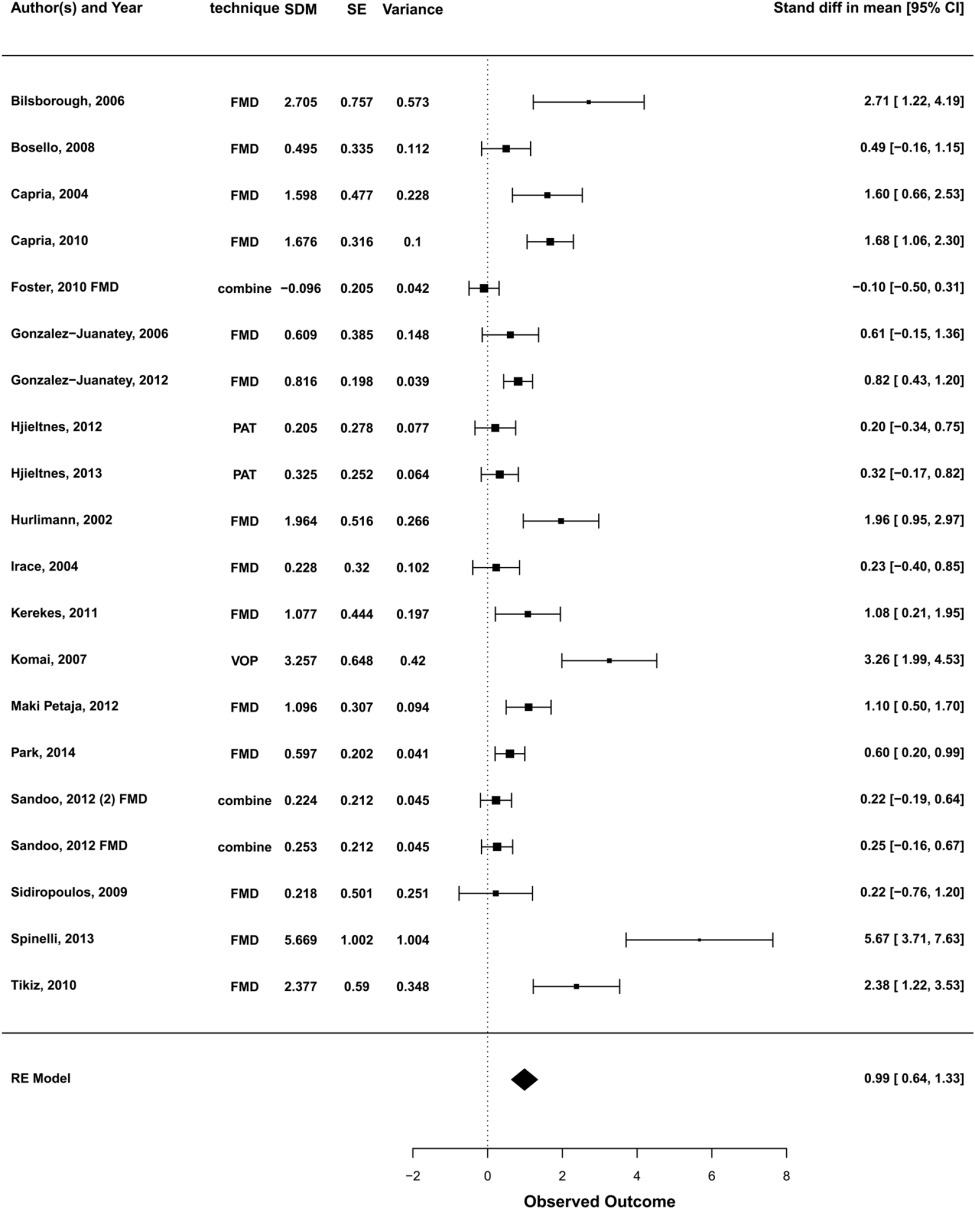
Forest plot for the effect of anti-TNF-α medications on measures of endothelial function. SDM, standardised difference in means; SE, standard error; CI, confidence interval; FMD, flow mediated dilatation; PAT, peripheral arterial tonometry; VOP, venous occlusion plethysmography; RE, random-effects. For those studies reporting more than one outcome for the same population and where the independence of values cannot be assumed, a combined outcome obtained by pooling FMD and LDI data was calculated in order to be more conservative regarding the precision of the point estimate.

The pooled analysis under a random-effects model demonstrated an estimated SDM of 0.987 (95%CI [0.64–1.33], p < 0.0001) (Fig. 2) suggesting an improvement of endothelial function after anti-TNF-α treatment.The null hypothesis that anti-TNF-α treatment had no effect (H0: µ = 0) could be clearly rejected (z = 5.65, p < 0.0001). Total heterogeneity was estimated to be τ^2^ = 0.451. The I^2^ statistic, which estimates the total variability in the effect size estimates which can be attributed to heterogeneity among the true effects, was calculated to be 82.7%. The H^2^ statistic (the ratio of the total amount of variability in the observed outcomes to the amount of sampling variability) was 5.77, revealing a degree of unexplained heterogeneity.

### Sensitivity analysis, subgroup analysis and random-effects meta-regression

As significant heterogeneity was observed, sensitivity analysis, subgroup analysis and random-effects meta-regression were performed to probe this issue further.

In one-study-removed sensitivity analysis, the pooled overall estimates were found to be stable after sequential exclusion of studies one at a time (Supplementary Fig. S5) suggesting a low susceptibility to outliers of the results.

**Figure 3:**
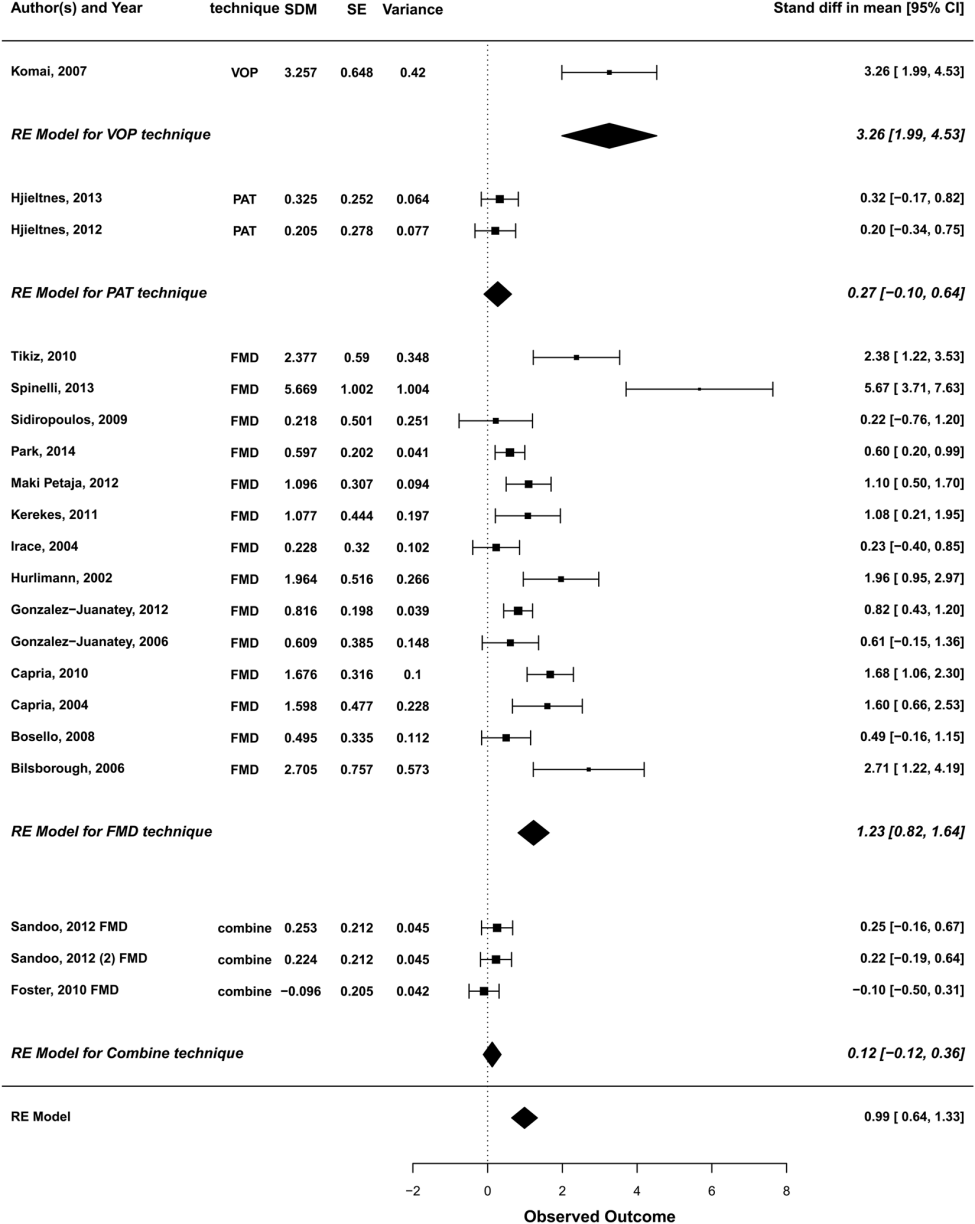
Subgroup analysis. A subgroup analysis was performed after stratification for technique used to assess endothelial function. Overall estimates for each subgroup were computed. SDM, standardised difference in means; SE, standard error; CI, confidence interval; FMD, flow mediated dilatation; PAT, peripheral arterial tonometry; VOP, venous occlusion plethysmography; RE, random-effects.

Next, we performed subgroup analysis after stratification according to the technique used to assess endothelial dysfunction (Fig. 3). Significant intra-group heterogeneity was observed in the FMD group (Q = 83.66, df = 16, p < 0.0001; I^2^ =  80.87%). In addition, we found a significant heterogeneity between groups (Q = 16.34, df = 3, p = 0.001).

A univariate random-effects meta-regression analysis was performed to evaluate the impact of potential moderators on the estimated effect size. The pooled analysis under the mixed-effects model using year as moderator gives an estimated amount of residual heterogeneity of τ^2^ = 0.4538, suggesting that −0.73% of the total amount of heterogeneity can be accounted for by including the year as moderator in the model. The omnibus test failed to reject the null hypothesis of no effect of the year on the effectiveness of the treatment (QM = 1.55, df = 1, p = 0.21). The test for residual heterogeneity was significant (QE = 104.2662, df = 18, p < 0.0001), possibly indicating that other moderators not considered in the model might influence the treatment effectiveness.

Technique is a factor variable with the method used in each study. We included 3 studies combining FMD and LDI, 14 studies using FMD, 2 studies using PAT and 1 studies using VOP. Different combination of the levels of ‘technique’ were explored: Model 1 (considering Combined technique, FMD, PAT, VOP separately), model 2 considering (FMD including Combined, PAT, VOP), model 3 considering FMD (including Combined), versus non-FMD (VOP and PAT). FMD and VOP were statistically significant in all the models. There was not enough evidence to detect any significant effect of Combined technique or PAT.

The pooled analysis under the mixed-effects model for each subgroup of technique gave an estimated amount of residual heterogeneity of τ^2^ = 0.2682, which suggests that 40.5% of the total amount of heterogeneity could be accounted for by including endothelial function technique as moderator in the model. Only FMD and VOP appeared to have a significant influence on the effect of anti-TNFα treatment on endothelial function.

### Publication bias

**Figure 4:**
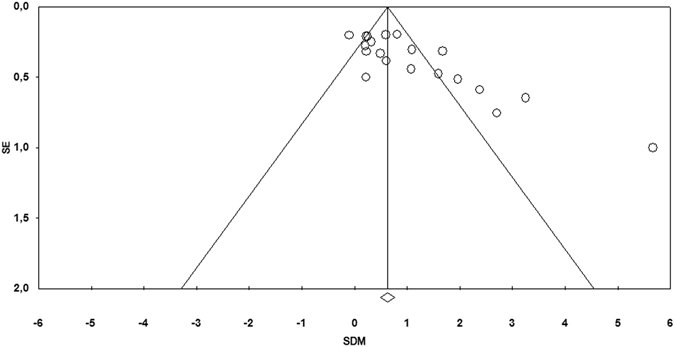
Funnel plot of standard error (SE) by standardised difference in means (SDM). A funnel plot obtained by plotting SE versus SDM demonstrates asymmetry to the right and therefore the presence of a potential publication bias.

Visual inspection of the funnel plot (Fig. 4) revealed a discrete asymmetry, and Egger’s regression test was performed to confirm the presence of significant publication bias. The intercept (B0) was 4.54 (95% CI [2.60–6.49]), with t = 4.92, df = 18, p = 0.0001. Subsequently, we assessed the funnel plot with the “trim and fill” method to evaluate the effect of publication bias on effect size results. Under the random-effects model, no studies were trimmed; therefore the imputed point estimate remained unchanged.

## Discussion

Rheumatoid arthritis has been largely associated with endothelial dysfunction, which, in turn, is now emerging as a factor possibly contributing to the overall risk of CVD events in RA patients^8, 9^. Consistently, most vascular diseases have been reported to be characterised by a preliminary damage to the vascular endothelium^13^. Over the last years, accumulating evidence suggests that anti-TNF-α therapy may reduce multiple CVD risk factors^17–20, 49^ including subclinical atherosclerosis and insulin resistance. Anti-TNF therapy has also been shown to reduce arterial stiffness and improve vascular wall physiology, with some evidence that this decreases progression of atherosclerosis^22^.

On this background, we performed a systematic review with meta-analysis to evaluate whether anti-TNF-α biologic treatment has a beneficial effect on endothelial dysfunction in RA patients. Results from our systematic literature search show that the evidence on this issue relies on observational and quasi-controlled studies of relatively low-quality and small-size, while no randomised controlled trials were available for inclusion in the review. Therefore, although providing information on surrogate measures pertinent with the outcome of interest, most of these studies were not adequately designed to investigate and catch a significant effect of anti-TNF-α treatment on endothelial function. Moreover, studies were highly heterogeneous, particularly with respect to sample size, medications allowed, comorbid diseases, characteristics of patients (i.e. RA duration), follow-up period, specific anti-TNF-α molecule administered and techniques for measuring endothelial function.

In our meta-analysis, the cumulative analysis demonstrated that anti-TNFα biologic treatment in RA patients led to a significant improvement in endothelial function. In particular the main meta-analysis effect was attributable to pooled analysis of FMD studies, since endothelial function technique emerged as a factor contributing to study heterogeneity. Improvement in endothelial function with anti-TNF-α therapy was also observed in a single study using VOP technique. Adjustment for study technique using a number of different models suggests that the conclusion of the overall meta-analysis remains unchanged, namely that anti-TNF-α therapy does appear to show an effect on improving endothelial function. On the one hand, removal of single studies as part of a sensitivity analysis suggested that the general meta-analysis conclusion was robust against single outlying studies. However the funnel plot (Fig. 4) demonstrated possible evidence of publication bias, and the present analysis cannot exclude the possibility that some negative studies remain unpublished, which could obviously negate the positive findings of the present systematic review.

The observation from the current systemic review is in contrast to one smaller and older review^50^ which failed to observe a significant effect of anti-TNF-α molecules on surrogate measures of arterial stiffness and endothelial dysfunction. This is most likely to be due to the inclusion in our study of more recent literature data and more comprehensive statistical analysis. Our finding is pathophysiologically complementary to those from two previously published meta-analyses^17, 18^, in particular to a pooled meta-analysis^18^ from 16 cohort studies showing a significant association between anti-TNF-α therapy and decreased risk of CVD events. Of note, both these reviews included clinical studies looking at hard cardiovascular outcomes; whereas our analysis focused on studies with physiological evaluation of endothelial function. Thus the present work represents the most comprehensive systematic literature review and meta-analysis on this topic and summarises the best evidence of the association between endothelial function and use of anti-TNF-α molecules in RA patients. More importantly, our cumulative meta-analysis confirmed several single, positive studies highlighting the potential of anti-TNF-α medications to improve vascular endothelial function ^29–32, 34–44, 47, 48, 51, 52^. In particular, Sandoo *et al*. reported that anti-TNF-α therapy improves microvascular endothelial function evaluated by LDI but not through FMD^53, 54^; this conflicting finding might be due to the intrinsic susceptibility of the FMD technique to a higher operator bias. On the other hand, anti-TNF-α therapy worsened endothelial function in only one^55^ of the reviewed studies. A very short follow-up duration (4 weeks) and extremely low pre-treatment (at baseline) FMD might account for such result. In accordance, worse baseline endothelial function could underlie the slight and non-significant improvement observed after anti-TNF-α treatment in two longer follow-up studies by Hjeltnes *et al*.^45, 46^. Taken together, these observations suggest that anti-TNF-α drugs might favourably impact endothelial dysfunction in the middle- to long-term in particular in those patients with relatively preserved endothelial function. In addition, the coexistence of multiple CVD risk factors (i.e. dyslipidaemia, smoking habit and T2DM) in patients may also justify the negative observation in the study by Foster *et al*.^55^ as compared with other reviewed studies.

Although the current meta-analysis provides overall evidence in support of the notion that that anti-TNF-α therapy improves endothelial function, the key limitation of the present review is the limited methodological quality of the included studies and primarily the lack of large scale randomised studies. All of the included studies were observational or quasi-controlled studies which may introduce bias in comparison to randomised controlled trials primarily designed to investigate the effect of anti-TNF-α treatment on endothelial function. However, it should be noted that conducting clinical trials aimed at exploring only the above surrogate endpoint would be difficult on ethical grounds. The variable follow-up period across studies also prevents us drawing useful conclusions on the durability of the observed effect. The presence of heterogeneity across studies also impacts on the generalizability of our findings to the whole RA population, since it raises the theoretical possibility that certain subgroups of RA patients may benefit more than others. Another unanswered issue is whether the effect on endothelial function is class-specific or simply related to a non-specific reduction in inflammatory burden. Some of the included studies ^30, 32, 42, 43^ described an inverse correlation between baseline endothelial function and measures of inflammation (erythrocyte sedimentation rate or C-reactive protein), but only one study^34^ demonstrated a direct correlation between change in C-reactive protein values after treatment and improvement in endothelial function. Additional evidence suggests that other medication, including synthetic-DMARDs^56^ and possibly other biologics^57^ may also improve endothelial function in RA patients.

In summary, our data suggests that there may be an identifiable, beneficial effect of anti-TNF-α therapy on improving endothelial dysfunction in RA patients. However adequately powered studies with larger sample size and longer follow-up duration, are required to fully dissect the relationship between anti-TNF-α therapy, endothelial dysfunction and actual cardiovascular events, in order to determine the long-term consequences of anti-TNF-α therapy on endothelial function and cardiovascular morbidity and mortality in individuals with RA.

## Electronic supplementary material

Supplementary Information
