# Supplementary material for: Anti-TNF-alpha agents and endothelial function in rheumatoid arthritis: a systematic review and meta-analysis
Source: Sci Rep. 2017 Jul 13;7:5346. doi: 10.1038/s41598-017-05759-2 (PMC5509678; doi:10.1038/s41598-017-05759-2)
Supplement: Supplementary file 1 — Supplementary Information [file 41598_2017_5759_MOESM1_ESM.doc]

**Anti-TNF alpha agents and endothelial function in rheumatoid arthritis: a systematic review and meta-analysis**

**Running title:** Anti-TNF alpha agents and endothelial function

**Francesco Ursini*^1^, Christian Leporini^1^, Fabiola Bene^3^, Salvatore D’Angelo^2^, Daniele Mauro^3^, Emilio Russo^1^, Giovambattista De Sarro^1^, Ignazio Olivieri^2^, Costantino Pitzalis^3^, Myles Lewis^3^ and Rosa Daniela Grembiale^1^**

^1^ Department of Health Sciences, University of Catanzaro “Magna Graecia”, Catanzaro, Italy

^2^ Rheumatology Department of Lucania, San Carlo Hospital of Potenza and Madonna delle Grazie Hospital of Matera, Potenza, Italy

^3^ William Harvey Research Institute and Barts and The London, School of Medicine and Dentistry Queen Mary University of London, London, United Kingdom.

**Corresponding author:**

Francesco Ursini, MD, PhD
Department of Health Sciences - University of Catanzaro “Magna Graecia”
viale Europa – 88100 Catanzaro, Italy (IT)

**Email:** francesco.ursini@yahoo.it
**Phone:** +39 0961.3694380 **Fax:** +39 0961369.4039

**KEYWORDS**

Rheumatoid arthritis, endothelial function, endothelial dysfunction, TNF alpha

**Supplementary Table S1.** Short description of the included techniques for assessing endothelial function.

| **Technique** | **Short description** |
| --- | --- |
| **Venous occlusion plethysmography (VOP)**[**^1^**](#_ENREF_1) | VOP is based on the principle that interruption of venous outflow from the forearm (leaving unaltered the arterial flow) leads to an increase in the forearm volume that is correlated with forearm vascular resistance, a measure of endothelial function. Changes in forearm volume are measured by plethysmography and medications can be given intra-arterially by placement of a fine needle into the brachial artery in order to study the local effect of vasoactive mediators in the forearm vascular bed. |
| **Flow-mediated dilatation (FMD)**[^2^](#_ENREF_2) | FMD measures the ability of the arteries to respond with endothelial nitric oxide (NO) release during flow-mediated reactive hyperaemia, after a 5-minute occlusion of the brachial artery with a blood pressure cuff. The measure of endothelial function is obtained by measuring the diameter of brachial artery before and after reactive hyperaemia with an ultrasound equipment. |
| **Peripheral arterial tonometry (PAT)**[^3^](#_ENREF_3) | PAT evaluates endothelium-mediated changes in the digital pulse waveform known as the PAT (peripheral arterial tone) signal, measured with a pair of plethysmographic probes situated on the index finger of each hand after creating a downstream hyperaemic response by occluding blood flow through the brachial artery for 5 minutes using an inflatable cuff. |
| **Laser Doppler iontophoresis (LDI)**[^4^](#_ENREF_4) | LDI involves the delivery of a vasoactive drug onto a patch of skin on the subjects forearm. The subsequent alterations in skin blood flow (related to microvascular endothelial function) are then detected using laser Doppler imaging. Acetylcholine and sodium nitroprusside are used to generate endothelium-dependent and endothelium-independent vasodilatation, respectively. |

**Supplementary Table S2.** Excluded studies after full-text evaluation.

| **Study author, year** | **Cause of exclusion** |
| --- | --- |
| Oranskyi et al., 2013[^5^](#_ENREF_5) | Full-text article available only in Russian language. |
| Watanabe et al., 2014[^6^](#_ENREF_6)  Galarraga et al., 2008[^7^](#_ENREF_7) | Cross-sectional design. |
| Mazzoccoli et al., 2010[^8^](#_ENREF_8) | Mixed population including both RA and psoriatic arthritis (PsA) patients. |
| Turiel et al., 2010[^9^](#_ENREF_9) | Endothelial function evaluated before and after synthetic DMARDs treatment. |
| Gonzalez-Juanatey et al., 2004[^10^](#_ENREF_10) | Patients already in treatment with anti-TNFα medications at study entry. |
| Gonzalez-Gay et al., 2006[^11^](#_ENREF_11)  Gonzalez-Gay et al., 2006 [^12^](#_ENREF_12)  Gonzalez-Gay et al., 2008[^13^](#_ENREF_13)  Di Franco et al., 2012[^14^](#_ENREF_14)  Sandoo et al., 2012[^15^](#_ENREF_15)  Meshkerina et al., 2015[^16^](#_ENREF_16) | Not evaluating one of the selected measures of endothelial function. |
| Hansel et al., 2003[^17^](#_ENREF_17) | No numerical data for endothelial function reported. |
| Cardillo et al., 2006[^18^](#_ENREF_18) | Endothelial function evaluated only before and immediately after anti-TNFα treatment. |

**Supplementary Table S3.** Additional characteristics of the included studies.

| **Study, year** | **Anti-TNFα molecule** | **Follow-up (months)** | **Age (years)** | **RA Duration (years)** | **DAS28** | **DMARDs** | **CCS** | **Statins** | **Smoke** | **T2DM** | **HBP** | **CAD** | **CKD** |
| --- | --- | --- | --- | --- | --- | --- | --- | --- | --- | --- | --- | --- | --- |
| Bilsborough et al., 2006[^19^](#_ENREF_19) | ETN  IFX | 36 | 56.6 ± 4.3 | - | 6.6 ± 0.5 | Y | Y | - | - | - | - | - | - |
| Bosello et al., 2008[^20^](#_ENREF_20) | IFX | 14 | 53.1 ± 7.8 | 12.8 ± 9.0 | 6.7 ± 1.0 | Y | Y | - | N | N | N | N | - |
| Capria et al., 2004[^21^](#_ENREF_21) | ETN IFX | 6 | 54.3 ± 8.7 | 8.7 ± 8 | 6.3 ± 1.6 | Y | Y | Y | Y | - | Y | - | - |
| Capria et al., 2010[^22^](#_ENREF_22) | ADA ETN  IFX | 6 | 49.8 ± 15.3 | 8.7 ± 8 | 6.3 ± 1.6 | Y | Y | Y | Y | - | Y | - | - |
| Foster et al., 2010[^23^](#_ENREF_23) | ADA  ETN  IFX | 4 | - | - | - | Y | Y | Y | Y | Y | Y | N | N |
| Gonzalez-Juanatey et al., 2006[^24^](#_ENREF_24) | ADA | 12 | 51 (24-74) | 20 (7-29) | 5.5 ± 1.3 | Y | Y | N | N | N | N | N | - |
| Gonzalez-Juanatey et al., 2012[^25^](#_ENREF_25) | ADA | 52 | 54.9 (47.5-63) | 4.9 | 5.9 ± 0.7 | Y | Y | Y | N | N | Y | N | - |
| Hjeltnes et al., 2012[^26^](#_ENREF_26) | ADA  ETN  IFX | 24 | 58 ± 8 | 9 ± 9 | 4.8 ± 1.2 | Y | Y | Y | Y | N | Y | Y | - |
| Hjeltnes et al., 2013[^27^](#_ENREF_27) | ADA  ETN  IFX | 24 | 58 ± 8 | 8 ± 8 | 5 | Y | Y | Y | Y | Y | Y | Y | - |
| Hurlimann et al., 2002[^28^](#_ENREF_28) | IFX | 12 | 46 ± 5 | 9 ± 2 | 5.6 ± 0.3 | Y | Y | - | Y | Y | Y | - | - |
| Irace et al., 2004[^29^](#_ENREF_29) | IFX | 6 | 46 ± 12 | 7 ± 2 | 3.4 ± 0.4 | Y | Y | N | - | - | - | - | - |
| Kerekes et al., 2011[^30^](#_ENREF_30) | ADA | 12 | 37.8 (24-69) | 5.6 (3-12) mo | 6.0 ± 0.77 | Y | N | - | N | N | N | N | N |
| Komai et al., 2007[^31^](#_ENREF_31) | IFX | 6 | 50 ± 3 | 10 ± 2.3 | 5.1 ± 0.2 | Y | Y | - | - | - | - | - | - |
| Maki-Petaja et al., 2012[^32^](#_ENREF_32) | ADA  ETN | 6 | 54 ± 19 | - | 6.1 ± 1.6 | Y | Y | - | N | N | N | N | N |
| Park, 2014[^33^](#_ENREF_33) | ADA  ETN | 24 | 55.2 ± 11.2 | 5.7 (3.3-10.7) | 5.6 (4.1-6.5) | Y | Y | Y | - | Y | Y | N | N |
| Sandoo et al. (1), 2012[^34^](#_ENREF_34) | ADA  ETN  IFX | 12 | 54 ± 15 | 11 ± 11 | 4.17 ± 1.0 | Y | Y | Y | Y | - | Y | N | - |
| Sandoo et al. (2), 2012[^35^](#_ENREF_35) | ADA  ETN  IFX | 12 | 54 ± 15 | 11 ± 11 | 4.2 ± 1.0 | Y | Y | Y | Y | - | Y | N | - |
| Sidiropoulos et al., 2009[^36^](#_ENREF_36) | ADA  IFX | 12 | 54.8 ± 15 | 13.3 ± 7.8 | 5.7 ± 0.7 | Y | N | - | - | - | - | N | - |
| Spinelli et al., 2013[^37^](#_ENREF_37) | ADA  ETN | 12 | 50.4 ± 24.4 | 8.6 ± 8.7 | 5.2 ± 1.1 | Y | Y | N | - | N | - | N | N |
| Tikiz et al., 2010[^38^](#_ENREF_38) | ETN | 12 | 47 ± 10.1 | 5.5 ± 3.3 | 4.8 ± 0.2 | Y | N | - | - | N | Y | N | N |

**Legend:** RA, rheumatoid arthritis; DAS28, disease activity score including 28 joints; DMARDs, disease-modifying anti-rheumatic drugs; CCS, corticosteroids; T2DM, type 2 diabetes mellitus; HBP, high blood pressure; CAD, coronary artery disease; CKD, chronic kidney disease; -, not reported, mo, months. Values are expressed as mean ± standard deviation or median (range).

**Supplementary Table S4.** Quality assessment* of included studies.

|  |  |  |  |  |  |  | **Item** |  |  |  |  |  |  |
| --- | --- | --- | --- | --- | --- | --- | --- | --- | --- | --- | --- | --- | --- |
|  | **1** | **2** | **3** | **4** | **5** | **6** | **7** | **8** | **9** | **10** | **11** | **12** | **Rate** |
| **Bilsborough et al.,** 2006[^19^](#_ENREF_19) | N | N | CD | CD | CD | Y | Y | NR | Y | Y | N | NA | Poor |
| **Bosello et al.,** 2008[^20^](#_ENREF_20) | Y | N | N | CD | CD | Y | Y | Y | Y | Y | Y | NA | Poor |
| **Capria et al.,** 2004[^21^](#_ENREF_21) | Y | N | N | CD | CD | Y | Y | NR | Y | N | Y | NA | Poor |
| **Capria et al.,** 2010[^22^](#_ENREF_22) | Y | N | N | CD | CD | Y | Y | NR | Y | N | Y | NA | Poor |
| **Foster et al.,** 2010[^23^](#_ENREF_23) | Y | Y | Y | CD | CD | Y | Y | Y | Y | Y | Y | NA | Fair |
| **Gonzalez-Juanatey et al.,** 2006[^24^](#_ENREF_24) | Y | N | Y | CD | CD | Y | Y | Y | Y | Y | Y | NA | Fair |
| **Gonzalez-Juanatey et al.,** 2012[^25^](#_ENREF_25) | Y | N | Y | CD | CD | Y | Y | Y | Y | Y | Y | NA | Fair |
| **Hjeltnes et al.,** 2012[^26^](#_ENREF_26) | Y | Y | Y | CD | CD | Y | Y | NR | Y | Y | Y | NA | Fair |
| **Hjeltnes et al.,** 2013[^27^](#_ENREF_27) | Y | Y | Y | CD | CD | Y | N | NR | Y | Y | Y | NA | Poor |
| **Hurlimann et al.,** 2002[^28^](#_ENREF_28) | Y | N | CD | CD | CD | N | Y | Y | Y | Y | N | NA | Fair |
| **Irace et al.,** 2004[^29^](#_ENREF_29) | N | N | CD | CD | CD | Y | Y | Y | Y | Y | Y | NA | Poor |
| **Kerekes et al.,** 2011[^30^](#_ENREF_30) | Y | N | CD | CD | CD | Y | Y | NR | Y | Y | Y | NA | Poor |
| **Komai et al.,** 2007[^31^](#_ENREF_31) | Y | N | CD | CD | CD | Y | Y | NR | Y | Y | Y | NA | Poor |
| **Maki-Petaja et al.,** 2012[^32^](#_ENREF_32) | Y | N | Y | CD | CD | Y | Y | NR | Y | Y | N | NA | Poor |
| **Park et al.,** 2014[^33^](#_ENREF_33) | Y | Y | Y | CD | CD | Y | Y | NR | Y | Y | N | NA | Poor |
| **Sandoo (1) et al.,** 2012[^34^](#_ENREF_34) | Y | N | Y | CD | CD | Y | Y | Y | Y | Y | Y | NA | Fair |
| **Sandoo (2) et al.,** 2012[^35^](#_ENREF_35) | Y | N | Y | CD | CD | Y | Y | Y | Y | Y | Y | NA | Fair |
| **Sidiropoulos et al.,** 2009[^36^](#_ENREF_36) | Y | N | CD | CD | CD | N | Y | Y | Y | Y | Y | NA | Poor |
| **Spinelli et al.,** 2013[^37^](#_ENREF_37) | Y | N | Y | CD | CD | Y | Y | Y | Y | Y | N | NA | Fair |
| **Tikiz et al.,** 2010[^38^](#_ENREF_38) | Y | N | CD | CD | CD | Y | Y | Y | Y | Y | N | NA | Poor |

**Table legend:** Y, yes; N, No; CD, cannot determine; NR, not reported; NA, not available

*Quality assessment was performed using the Quality Assessment Tool for Before-After (Pre-Post) Studies With No Control Group proposed by the National Heart, Lung, and Blood Institute - US Department of Health & Human Services (<https://www.nhlbi.nih.gov/health-pro/guidelines/in-develop/cardiovascular-risk-reduction/tools/before-after>).

**Supplementary Figure S5. One-study-removed sensitivity analysis.** The pooled overall estimates were calculated after sequential exclusion of studies one at a time. SE, standard error; CI, confidence interval; FMD, flow mediated dilatation; LDI, laser-Doppler iontophoresis; PAT, peripheral arterial tonometry; VOP, venous occlusion plethysmography.


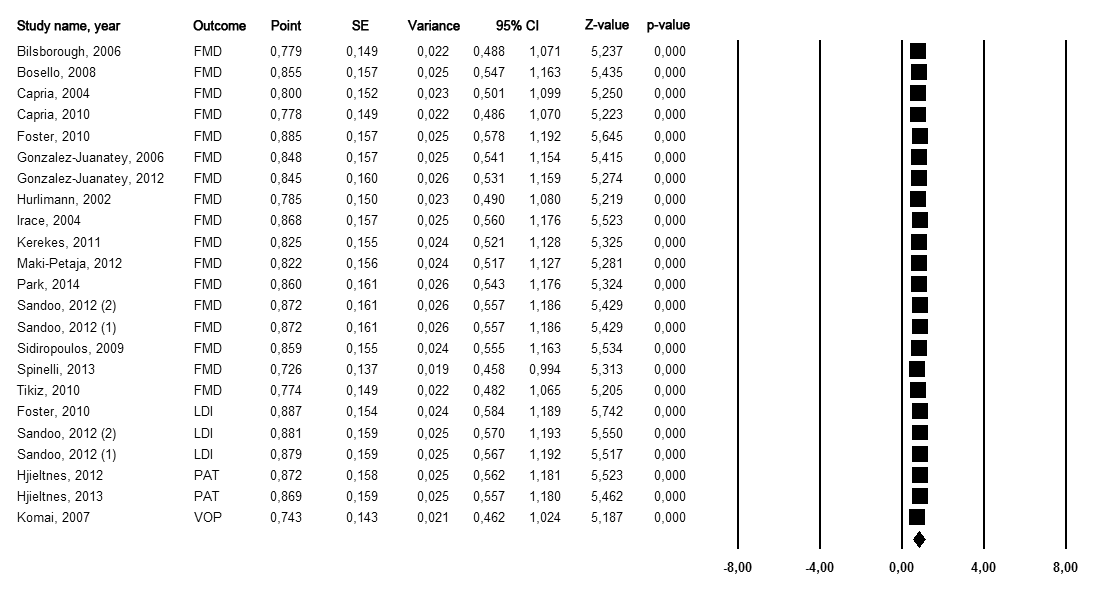


**References**

1 Linder, L., Kiowski, W., Buhler, F. R. & Luscher, T. F. Indirect evidence for release of endothelium-derived relaxing factor in human forearm circulation in vivo. Blunted response in essential hypertension. *Circulation* **81**, 1762-1767 (1990).

2 Celermajer, D. S. *et al.* Non-invasive detection of endothelial dysfunction in children and adults at risk of atherosclerosis. *Lancet* **340**, 1111-1115 (1992).

3 Kuvin, J. T. *et al.* Assessment of peripheral vascular endothelial function with finger arterial pulse wave amplitude. *Am Heart J* **146**, 168-174, doi:10.1016/S0002-8703(03)00094-2

S0002870303000942 [pii] (2003).

4 Ramsay, J. E., Ferrell, W. R., Greer, I. A. & Sattar, N. Factors critical to iontophoretic assessment of vascular reactivity: implications for clinical studies of endothelial dysfunction. *J Cardiovasc Pharmacol* **39**, 9-17 (2002).

5 Oranskyi SP, E. L., Samorodskaya NA, Malkhasyan IG. Dynamics of cardiovascular status and serum markers of endothelial dysfunction in patients with rheumatoid arthritis, treated with infliximab. *Cardiovascular Therapy and Prevention (Russian Federation)* **12** (2013).

6 Watanabe, T. *et al.* Clinical significance of brachial flow-mediated dilation in patients with rheumatoid arthritis. *Int J Rheum Dis* **17**, 26-33, doi:10.1111/1756-185x.12021 (2014).

7 Galarraga, B., Khan, F., Kumar, P., Pullar, T. & Belch, J. J. F. C-reactive protein: The underlying cause of microvascular dysfunction in rheumatoid arthritis. *Rheumatology* **47**, 1780-1784, doi:10.1093/rheumatology/ken386 (2008).

8 Mazzoccoli, G. *et al.* Anti-tumor necrosis factor-alpha therapy and changes of flow-mediated vasodilatation in psoriatic and rheumatoid arthritis patients. *Intern Emerg Med* **5**, 495-500, doi:10.1007/s11739-010-0458-6 (2010).

9 Turiel, M. *et al.* Effects of long-term disease-modifying antirheumatic drugs on endothelial function in patients with early rheumatoid arthritis. *Cardiovascular Therapeutics* **28**, e53-e64, doi:10.1111/j.1755-5922.2009.00119.x (2010).

10 Gonzalez-Juanatey, C. *et al.* Active but transient improvement of endothelial function in rheumatoid arthritis patients undergoing long-term treatment with anti-tumor necrosis factor alpha antibody. *Arthritis Rheum* **51**, 447-450, doi:10.1002/art.20407 (2004).

11 Gonzalez-Gay, M. A. *et al.* Influence of anti-TNF-alpha infliximab therapy on adhesion molecules associated with atherogenesis in patients with rheumatoid arthritis. *Clin Exp Rheumatol* **24**, 373-379 (2006).

12 Gonzalez-Gay, M. A. *et al.* Anti-tumor necrosis factor-alpha blockade improves insulin resistance in patients with rheumatoid arthritis. *Clin Exp Rheumatol* **24**, 83-86 (2006).

13 Gonzalez-Gay, M. A. *et al.* Anti-tumour necrosis factor α therapy modulates ghrelin in patients with severe rheumatoid arthritis. *Annals of the Rheumatic Diseases* **67**, 1644-1646, doi:10.1136/ard.2008.088773 (2008).

14 Di Franco, M. *et al.* Serum levels of asymmetric dimethylarginine and apelin as potential markers of vascular endothelial dysfunction in early rheumatoid arthritis. *Mediators Inflamm* **2012**, 347268, doi:10.1155/2012/347268 (2012).

15 Sandoo, A. *et al.* Clinical remission following treatment with tumour necrosis factor-alpha antagonists is not accompanied by changes in asymmetric dimethylarginine in patients with rheumatoid arthritis. *Clinical Biochemistry* **45**, 1399-1403, doi:10.1016/j.clinbiochem.2012.07.092 (2012).

16 Meshcherina, N. S., Knyazeva, L. A., Goryainov, I. I. & Knyazeva, L. I. Vasoprotective effects of genetically engineered biologic drugs in patients with rheumatoid arthritis. *Sovremennye Tehnologii v Medicine* **7**, 130-136, doi:10.17691/stm2015.7.3.18 (2015).

17 Hänsel, S., Lässig, G., Pistrosch, F. & Passauer, J. Endothelial dysfunction in young patients with long-term rheumatoid arthritis and low disease activity. *Atherosclerosis* **170**, 177-180, doi:10.1016/s0021-9150(03)00281-8 (2003).

18 Cardillo, C. *et al.* Intravascular tumor necrosis factor alpha blockade reverses endothelial dysfunction in rheumatoid arthritis. *Clin Pharmacol Ther* **80**, 275-281, doi:10.1016/j.clpt.2006.05.011 (2006).

19 Bilsborough, W. *et al.* Anti-tumour necrosis factor-alpha therapy over conventional therapy improves endothelial function in adults with rheumatoid arthritis. *Rheumatology International* **26**, 1125-1131, doi:10.1007/s00296-006-0147-y (2006).

20 Bosello, S. *et al.* TNF-alpha blockade induces a reversible but transient effect on endothelial dysfunction in patients with long-standing severe rheumatoid arthritis. *Clin Rheumatol* **27**, 833-839, doi:10.1007/s10067-007-0803-y (2008).

21 Capria, A. *et al.* Endothelial dysfunction in rheumatoid arthritis is improved by anti-tumor necrosis factorα treatment. *European Journal of Inflammation* **2**, 113-118 (2004).

22 Capria, A. *et al.* Long-term anti-TNF-α treatments reverse the endothelial dysfunction in rheumatoid arthritis: The biological coherence between synovial and endothelial inflammation. *International Journal of Immunopathology and Pharmacology* **23**, 255-262 (2010).

23 Foster, W., Carruthers, D., Lip, G. Y. H. & Blann, A. D. Inflammation and microvascular and macrovascular endothelial dysfunction in rheumatoid arthritis: Effect of treatment. *Journal of Rheumatology* **37**, 711-716, doi:10.3899/jrheum.090699 (2010).

24 Gonzalez-Juanatey, C. *et al.* Short-term adalimumab therapy improves endothelial function in patients with rheumatoid arthritis refractory to infliximab. *Clinical and Experimental Rheumatology* **24**, 309-312 (2006).

25 Gonzalez-Juanatey, C. *et al.* Anti-TNF-alpha-adalimumab therapy is associated with persistent improvement of endothelial function without progression of carotid intima-media wall thickness in patients with rheumatoid arthritis refractory to conventional therapy. *Mediators Inflamm* **2012**, 674265, doi:10.1155/2012/674265 (2012).

26 Hjeltnes, G. *et al.* Relations of serum COMP to cardiovascular risk factors and endothelial function in patients with rheumatoid arthritis treated with methotrexate and TNF-α inhibitors. *Journal of Rheumatology* **39**, 1341-1347, doi:10.3899/jrheum.111401 (2012).

27 Hjeltnes, G. *et al.* Serum levels of lipoprotein(a) and E-selectin are reduced in rheumatoid arthritis patients treated with methotrexate or methotrexate in combination with TNF-α-inhibitor. *Clinical and Experimental Rheumatology* **31**, 415-421 (2013).

28 Hürlimann, D. *et al.* Anti-tumor necrosis factor-α treatment improves endothelial function in patients with rheumatoid arthritis. *Circulation* **106**, 2184-2187, doi:10.1161/01.cir.0000037521.71373.44 (2002).

29 Irace, C. *et al.* Effect of anti TNFalpha therapy on arterial diameter and wall shear stress and HDL cholesterol. *Atherosclerosis* **177**, 113-118, doi:10.1016/j.atherosclerosis.2004.04.031 (2004).

30 Kerekes, G. *et al.* Effects of adalimumab treatment on vascular disease associated with early rheumatoid arthritis. *Israel Medical Association Journal* **13**, 147-152 (2011).

31 Komai, N., Morita, Y., Sakuta, T., Kuwabara, A. & Kashihara, N. Anti-tumor necrosis factor therapy increases serum adiponectin levels with the improvement of endothelial dysfunction in patients with rheumatoid arthritis. *Modern Rheumatology* **17**, 385-390, doi:10.1007/s10165-007-0605-8 (2007).

32 Maki-Petaja, K. M. *et al.* Anti-tumor necrosis factor-alpha therapy reduces aortic inflammation and stiffness in patients with rheumatoid arthritis. *Circulation* **126**, 2473-2480, doi:10.1161/CIRCULATIONAHA.112.120410

CIRCULATIONAHA.112.120410 [pii] (2012).

33 Park, Y. J. *et al.* Bone erosion is associated with reduction of circulating endothelial progenitor cells and endothelial dysfunction in rheumatoid arthritis. *Arthritis and Rheumatology* **66**, 1450-1460, doi:10.1002/art.38352 (2014).

34 Sandoo, A., Kitas, G. D., Carroll, D. & Veldhuijzen van Zanten, J. J. C. S. The role of inflammation and cardiovascular disease risk on microvascular and macrovascular endothelial function in patients with rheumatoid arthritis: A cross-sectional and longitudinal study. *Arthritis Research and Therapy* **14**, doi:10.1186/ar3847 (2012).

35 Sandoo, A., Van Zanten, J. J. C. S. V., Toms, T. E., Carroll, D. & Kitas, G. D. Anti-TNFα therapy transiently improves high density lipoprotein cholesterol levels and microvascular endothelial function in patients with rheumatoid arthritis: A Pilot Study. *BMC Musculoskeletal Disorders* **13**, doi:10.1186/1471-2474-13-127 (2012).

36 Sidiropoulos, P. I. *et al.* Sustained improvement of vascular endothelial function during anti-TNFα treatment in rheumatoid arthritis patients. *Scandinavian Journal of Rheumatology* **38**, 6-10, doi:10.1080/03009740802363768 (2009).

37 Spinelli, F. R. *et al.* Effect of therapeutic inhibition of TNF on circulating endothelial progenitor cells in patients with rheumatoid arthritis. *Mediators of Inflammation* **2013**, doi:10.1155/2013/537539 (2013).

38 Tikiz, H., Arslan, Ö., Pirildar, T., Tikiz, C. & Bayindir, P. The effect of anti-tumor necrosis factor (TNF)-alpha therapy with etanercept on endothelial functions in patients with rheumatoid arthritis. *Anadolu Kardiyoloji Dergisi* **10**, 98-103, doi:10.5152/akd.2010.031 (2010).
